# Three-Layer Heterogeneous Network Combined With Unbalanced Random Walk for miRNA-Disease Association Prediction

**DOI:** 10.3389/fgene.2019.01316

**Published:** 2020-01-10

**Authors:** Limin Yu, Xianjun Shen, Duo Zhong, Jincai Yang

**Affiliations:** ^1^ School of Computer, Central China Normal University, Wuhan, China; ^2^ Hubei Provincial Key Laboratory of Artificial Intelligence and Smart Learning, Central China Normal University, Wuhan, China

**Keywords:** miRNA-disease association prediction, three-layer heterogeneous network, unbalanced random walk, *LncRNA*, Laplace normalization

## Abstract

miRNA plays an important role in many biological processes, and increasing evidence shows that miRNAs are closely related to human diseases. Most existing miRNA-disease association prediction methods were only based on data related to miRNAs and diseases and failed to effectively use other existing biological data. However, experimentally verified miRNA-disease associations are limited, there are complex correlations between biological data. Therefore, we propose a novel Three-layer heterogeneous network Combined with unbalanced Random Walk for MiRNA-Disease Association prediction algorithm (TCRWMDA), which can effectively integrate multi-source association data. TCRWMDA based not only on the known miRNA—disease associations, also add the new priori information (lncRNA–miRNA and lncRNA–disease associations) to build a three-layer heterogeneous network, lncRNA was added as the transition path of the intermediate point to mine more effective information between networks. The AUC value obtained by the TCRWMDA algorithm on 5-fold cross validation is 0.9209, compared with other models based on the same similarity calculation method, TCRWMDA obtained better results. TCRWMDA was applied to the analysis of four types of cancer, the results proved that TCRWMDA is an effective tool to predict the potential miRNA-disease association. The source code and dataset of TCRWMDA are available at: https://github.com/ylm0505/TCRWMDA.

## Introduction

MiRNAs are widely found in eukaryotes and regulate the expression of other genes. miRNA is very important for the control of animal development and physiology (Victor, 2004). miRNA is involved in regulating cell differentiation (Lee et al., 1993)and plays an important role in many biological processes, including cell cycle progression and apoptosis (Brennecke et al., 2003). Mutations and biogenic dysfunction of miRNA and disorders of miRNA and its targets may lead to a variety of diseases. Calin et al. published the first study that microRNAs linked to cancer in 2002, there was a significant association between decreased levels of both miRNAs and chronic lymphoblastic leukemia, suggesting a potential relationship between miRNA and cancer (Calin et al., 2002). miRNA is an important factor in tumorigenesis, and the artificial regulation of some miRNAs may lead to the occurrence or apoptosis of tumors, which depends on the regulation of miRNA (Yang et al., 2009). With the development of miRNA research, the association between miRNA and disease has been extended to many types of cancer, including leukemia and lung cancer (Johnson et al., 2005; Bandyopadhyay et al., 2010), breast cancer, and colon cancer (Michael et al., 2003), and so on, exploring the relationship between miRNA and disease has become the subject of many kinds of cancer research. More and more evidence proving that miRNA is closely related to diseases, understanding relationships between miRNA and disease is conducive to understanding the pathogenesis of diseases at the molecular level, but more importantly is conducive to prognosis, diagnosis, evaluation, treatment, and prevention of diseases and the promotion of human medical progress. Traditional experiments are costly, time consuming, and only suitable for small-scale data, with the development of biology, mass biological data about miRNA have been generated. There is an urgent need to develop a powerful computational method to predict the potential disease-related miRNAs, possible candidate miRNAs with higher prediction score were obtained by computational methods can reduce the time and cost of biological experiment.

In the early research methods of miRNA-disease association prediction, under the assumption that functionally related miRNAs are often related to diseases with similar phenotypes (Lu et al., 2008), A computational model based on hypergeometric distribution to predict the miRNA-disease association was proposed (Jiang et al., 2010), and constructed a heterogeneous phenome-microRNAome network for human phenome-microRNAome by combining the miRNA functional similarity network and the disease phenotype similarity network with the known miRNA-disease association, However, this method relies on the neighbor point information of the predicted miRNA, and the false positive and false negative rates are relatively high, so the prediction accuracy of this method is not high. With the development of miRNA-disease research, the restart random walk algorithm was used to predict the miRNA-disease association (RWRMDA) based on the similarity model, which is the first to use the global network to predict miRNA-disease association (Chen et al., 2012b). A restart random walk was performed on the MiRNA functional similarity network to predict potential MiRNA disease interactions, but RWRMDA did not work on any known related MiRNA disease. A semi-supervised classification method RLSMDA to predict the potential miRNA-disease association based on regularized least squares is proposed (Chen and Yan, 2015), RLSMDA is a semi-supervised model that does not require negative samples and a global approach that prioritizing the association of all diseases at the same time. Combined Within-Score with Between-Score for miRNA-disease association prediction (WBSMDA) was proposed (Chen et al., 2016), WBSMDA based on the basis of known miRNA-disease association data and assuming that miRNAs with similar functions are more likely to be associated with diseases with similar phenotypes may lead to bias (preference) on miRNAs with more known diseases, In addition, the accuracy of the model is still not very high. Then, a KNN model based on rank to predict potential related miRNAs for diseases (RKNNMDA) was proposed (Chen et al., 2017), which based on miRNA functional similarity, disease semantic similarity, Gaussian interaction profile kernel similarity and known miRNA-disease association. In RKNNMDA, k-nearest neighbor algorithm was used to search k-nearest neighbor of miRNA and disease, and these k-nearest neighbors were reordered and reweighted according to the support vector machine model to obtain the final predicted results. Random walk has also been further developed in the prediction of miRNA-disease association. The random walk technique has also been developed in association prediction, unbalanced bi-random walk on the heterogeneous networks (BRWH) based on RWR was proposed (Luo and Xiao, 2017) to predict the miRNA-disease Association. From the matrix, making use of matrix completion algorithm (MCMDA) to update the adjacency matrix based on the known miRNA-disease association data to predict its potential association proposed in (Li et al., 2017). In 2018, there is a KATZMDA model for miRNA-disease association prediction (Qu et al., 2018), which based on KATZ model to calculate miRNA similarity and disease similarity to predict the association between miRNA and disease, and KATZMDA yields better results than the previous algorithms mentioned. Based on the idea of MCMDA, a new induction matrix completion model (IMCMDA) for MiRNA-Disease Association prediction was proposed (Chen et al., 2018). Different from MCMDA, IMCMDA uses disease similarity and miRNA similarity as the characteristics of disease and miRNA to complete the missing miRNA-disease association. Recently, a kernel-based soft-neighborhood similarity model combined with similar network fusion for miRNA-disease association prediction was proposed (Ma et al., 2018a). The improvement of the similarity model improves the accuracy of predicting miRNA-disease. Ha et al. predict miRNA and disease associations based on matrix decomposition, which has been widely used in recommendation systems (Ha et al., 2019). Based on the heterogeneous network of miRNA and disease, structural perturbation method is also applied to the prediction of miRNA-disease correlation, and the final perturbed matrix represents the correlation score between the two (Zeng et al., 2018). However, these methods mentioned above only considered the miRNA-disease association data sets and functional similarity, without extracting more information from other data sets related to them to improve the accuracy and reliability of the model.

With the development of biomedicine, the number of biological databases increases, and the association between biological data is gradually excavated, which enables us to combine different information from different databases to reliably predict the miRNA-disease association. In view of the limitations of the above methods, in this paper, we put forward a novel prediction model of three-layer network combining unbalanced random walk for miRNA-disease association prediction (TCRWMDA). Based on the known associated data of miRNA-disease, lncRNA–miRNA and lncRNA-disease, TCRWMDA build a three-layer heterogeneous network and performs unbalanced random walk between networks and on heterogeneous networks to obtain the final prediction results.

To evaluate the effectiveness of the TCRWMDA, we compared it with other classical and advanced algorithms based on the same similarity measure on 5-fold cross-validation. In addition, compared with the latest model based on the kernel-based soft neighborhood network fusion similarity model. In order to verify the applicability of TCRWMDA algorithm, four diseases were studied by TCRWMDA algorithm. Experimental results and case studies show that this method can be effectively used to predict the potential association between miRNA and disease.

## Materials and Methods

### The Dataset

The associated data sets used in this article are from (Chen, 2015). The dataset mainly consists of three association data sets. First, miRNA-disease association data set is from HMDDV2.0 (Li et al., 2013), finally, 5,430 miRNA-disease associations were obtained, including 383 diseases and 495 miRNAs. *A* represents the known association between miRNA and disease, *A*(*i*,*j*)=1. denotes miRNA *m*(*i*) is related to disease *d*(*j*), otherwise, *A*(*i*,*j*)=0.

$$A(i,j)=\{\begin{array}{c} 1,if\;miRNA\;m(i)\;is\;associated\;with\;lncRNA\;l(j)\\ 0,otherwise \end{array}$$

Second, the lncRNA–miRNA association dataset was derived from the star-base v2.0 database (Yang et al., 2011). Repeated associations of different evidences were deleted, as well as the lncRNA–miRNA associations that did not exist in 5,430 known miRNA-disease associations and their corresponding lncRNA–miRNA associations in the lncRNA-disease association. Finally, 704 lncRNA–miRNA associations were obtained. *B* represents the known relationship between lncRNA–miRNA, *B*(*i*,*j*)=1 represents miRNA *m*(*i*) is related to lncRNA *l*(*j*),otherwise, *B*(*i,j*)=0.

$$B(i,j)=\{\begin{array}{c} 1,if\;miRNA\;m(i)\;is\;associated\;with\;\mathrm{IncRNA}\;I(j)\\ 0,otherwises \end{array}$$

Third, the lncRNA-disease association data set in the lncRNA Disease database (Geng Chen et al., 2012a) was downloaded, and the repeated association of different evidences and the association of lncRNA-disease related to the disease or lncRNA were removed. After removing the data of diseases not shown in the above data set, 182 lncRNA-disease associations of 34 lncRNAs were finally obtained. *C* represents association matrix between lncRNA and disease, *C*(*i*,*j*)=1 denotes lncRNA *l*(*i*) is related to disease *d*(*j*), otherwise, *C*(*i*,*j*)=0.

$$C(i,j)=\{\begin{array}{c} 1,if\;\mathrm{lncRNA}\;l(i)\;associated\;with\;disease\;d(j)\\ 0,otherwise \end{array}$$

### TCRWMDA

**Figure 1:**
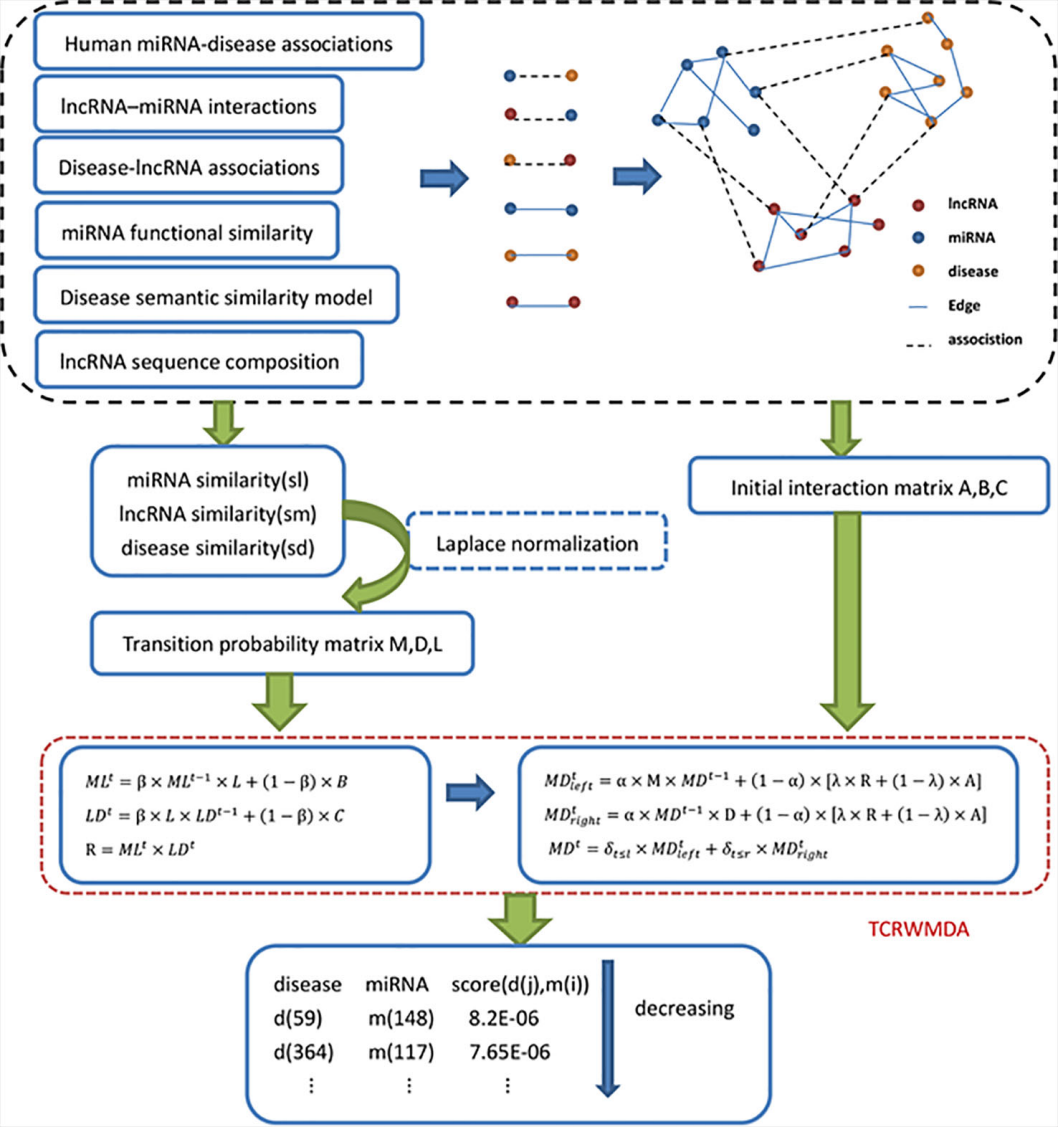
Flow chart of TCRWMDA algorithm. The steps of TCRWMDA for the association prediction between miRNA and disease are divided into four stages: the construction of similarity network, the calculation of transition probability matrix and the random walk on the three-layer heterogeneous network. Finally, the final prediction score is obtained to analyze the association probability of a certain disease and a certain miRNA. In the black dotted box is the construction of similarity network, which are based on association data and related data from the available database. The red dotted line shows that an unbalanced random walk on a three-layer heterogeneous network.

Based on the idea of unbalanced bi-random walk, we proposed three-layer heterogeneous network combined with unbalanced random walk for miRNA-disease association prediction algorithm. TCRWMDA algorithm includes three random walks, including the random walk on miRNA–miRNA network, disease similarity network, and the mapping relationship of miRNA–lncRNA-disease. **Figure 1** shows the flow chart of TCRWMDA algorithm to predict miRNA-disease association. In the dotted black box above **Figure 1**, blue dots represent miRNA, yellow dots represent disease, and red dots represent lncRNA. A three-layer heterogeneous network consist of the similar networks formed by same color nodes with straight lines and the heterogeneous networks formed by nodes of different colors with dotted lines. The similarity measure can be obtained by calculating the similarity of association data, the similarity measure was use to obtain the transition probability matrix by Laplace normalization, finally, TCRWMDA algorithm using the transition probability matrix to unbalanced random walk on heterogeneous network to get the potential association scores between the disease and its associated miRNAs and sorting. The feasibility and effectiveness of the algorithm is verified by whether the predicted results already exist in the existing database.

#### Construction of Similarity Networks

The similarity networks in this paper consist of lncRNA similarity network, Disease similarity network, miRNA similarity network.

##### lncRNA Similarity Network

Genes can be mutated, inserted and deleted, it is difficult to achieve a complete match of two sequences, so we use sequence information as its feature. We extract the sequence features by considering sequence composition (Zhang et al., 2018). For lncRNA sequences, we calculated the proportion of four nucleotide types (A, C, G, T) and 16 dinucleotide types (AA, AG, AC…) in each lncRNA sequence, every lncRNA *l*(*i*) can get a 20−dimensional eigenvector, where (*i*) is its component, named as lncRNA sequence composition. The sequence data of 34 selected lncRNA were downloaded from LNCipedia5 (Volders et al., 2019). Use cosine similarity method to calculate the lncRNA similarity *sl*, the formula of lncRNA similarity is as follows:

$$sl\left(i,j\right)=\frac{\sum_{i=1}^{20}ℒ\left(i\right)\times ℒ\left(j\right)}{\sqrt{\sum_{i=1}^{20}{\left(ℒ\left(i\right)\right)}^{2}}\times \sqrt{\sum_{j=1}^{20}{\left(ℒ\left(j\right)\right)}^{2}}}$$

##### Disease Similarity Network

In this paper, we used the same method as in literature (Wang et al., 2010) to calculate the disease similarity.

Disease semantic similarity model 1: Directed acyclic graph (DAG) was constructed to describe the disease based on MeSH descriptor downloaded from national library of medicine (Lipscomb, 2000) (http://www.nlm.nih). According to DAG, the contribution of disease *d* to the semantic value of disease *d* DAG (d) is expressed as:

$$\{\begin{array}{cc} D1_{D}\left(d\right)=1 & if\;d=D\\ D1_{D}\left(d\right)=\left\{\Delta *D1_{D}\left(d^{\prime }\right)|d^{\prime }\in children\;of\;d\right\} & if\;d\neq D \end{array}$$

∆ denotes attenuation coefficient of semantic contribution. The self-semantic value of disease D was defined as follows:

$$\text{DV}1\left(\text{D}\right)=\sum_{d\in T\left(D\right)}D1_{D}\left(d\right)$$

Where T(D) represents all ancestor nodes of D and D itself. Based on the assumption that the two diseases share a large part of DAG and their semantic similarity is large, the semantic similarity between disease *d*(*i*). and disease *d*(*j*) can be defined as:

$$\text{SS}1\left(d\left(i\right),d\left(j\right)\right)=\frac{\sum_{d\in T\left(d\left(i\right)\right)\cap T\left(d\left(j\right)\right)}\left(D1_{d\left(i\right)}\left(d\right)+D1_{d\left(j\right)}\left(d\right)\right)}{\text{DV}1\left(d\left(i\right)\right)+\text{DV}1\left(d\left(j\right)\right)}$$

Disease semantic similarity model 2: It is unreasonable to give the same contribution value for diseases in the same layer of DAG (D). Therefore, according to the model proposed by Xuan et al., we define the contribution of disease d to the semantic value of disease d in DAG (d) as follows:

$$D2_{D}\left(d\right)=-\log\left[\frac{the\;number\;of\;DAGs\;including\;d}{the\;number\;of\;diseases}\right]$$

We define the semantic similarity of diseases *d*(*i*),*d*(*j*) as the ratio of share ancestor node contributions to all ancestor node contributions. The semantic similarity model 2 is calculated as follows:

$$\text{SS}2\left(d\left(i\right),d\left(j\right)\right)=\frac{\sum_{d\in T\left(d\left(i\right)\right)\cap^{}T\left(d\left(j\right)\right)}\left(D2_{d\left(i\right)}\left(d\right)+D2_{d\left(j\right)}\left(d\right)\right)}{\text{DV}2\left(d\left(i\right)\right)+\text{DV}2\left(d\left(j\right)\right)}$$

Among them,

$$\text{DV}2\left(\text{D}\right)=\sum_{d\in T\left(D\right)}D2_{D}\left(d\right)$$

##### miRNA Similarity Network

Wang et al. (2010) proposed the method of MISIM and miRNA functional similarity based on the hypothesis that miRNAs with similar functions are more likely to be associated with diseases with similar characteristics. The miRNA function similarity data downloaded from http://www.cuilab.cn/files/images/cuilab/misim.zip. We use *FS*(*m*(*i*),*m*(*j*)) to represent association score between miRNA *m*(*i*) and miRNA *m*(*j*)

##### Gaussian Kernel Similarity

Based on the basic assumption that similar diseases are often associated with miRNAs with similar functions (Wang et al., 2010), we calculated the Gaussian kernel similarity for miRNA and disease to obtain the miRNA similarity and disease similarity. First, we use vector *IP*(*d*(*i*)) to represent there is or is not an association between each miRNA and disease *d*(*i*) and regard *IP*(*d*(*i*)) as interaction profile of the disease *d*(*i*), then, the gaussian interaction profile kernel similarity between disease *d*(*i*) and *d*(*j*) was calculated:

$$kd\left(i,j\right)=exp\left(-\gamma_{d}{\left\|IP\left(d\left(i\right)\right)-IP(d\left(j\right))\right\|}^{2}\right)$$

$$\gamma_{d}={\gamma^{\prime }}_{d}/(\frac{1}{nd}\sum_{i=1}^{nd}\left||IP(d(i)\right))|{|}^{2})$$

*γ_d_* controls kernel bandwidth. Similarly, the Gaussian kernel similarity between disease *m*(*i*) and disease *m*(*j*) can be obtained as follows:

$$km\left(i,j\right)=exp\left(-\gamma_{m}{\left\|IP\left(m\left(i\right)\right)-IP(m\left(j\right))\right\|}^{2}\right)$$

$$\gamma_{m=}{\gamma^{\prime }}_{m}/(\frac{1}{nm}\sum_{i=1}^{nm}||IP(m(i))|{|}^{2})$$

##### Integrated Similarity for Diseases and miRNAs

We could not obtain the DAGs of all diseases, that is, for a specific disease without DAG, we could not calculate the semantic similarity score of this disease with other diseases. Therefore, for the disease pairs with semantic similarity score, we used the semantic similarity score to express the disease similarity, and for other disease pairs, we used the gaussian kernel interaction profile similarity to represent the disease similarity. The disease similarity matrix of disease *d*(*i*) and disease *d*(*j*) was constructed as follows:

$$sd\left(i,j\right)=\{\begin{array}{c} \frac{SS1\left(d\left(i\right),d\left(j\right)\right)+SS2\left(d\left(i\right),d\left(j\right)\right)}{2}\;d\left(i\right)\;and\;d\left(j\right)\;has\;semantic\\ similarity\\ kd\left(d\left(i\right),d\left(j\right)\right)\;\;\;\;\;\;\;\;\;\;\;\;\;\;\;\;\;otherwise \end{array}$$

Similarly, the similarity matrix of miRNA can be obtained:

$$sm(i,j)=\{\begin{array}{cc} FS(m(i),m(j))\;m(i)\;and\;m(j)\;has\;functional\;similarity & \\ km(m(i),m(j)) & otherwise \end{array}$$

The similarity between the two miRNAs is the weight of edge in the miRNA similarity network, in the same way, the similarity between the two diseases is the weight of edge in the disease similarity network.

#### Calculation of Transition Probability Matrix

To perform a random walk on three-layer heterogeneous networks, the state transition between networks must be considered and transition probability matrix needs to be created. To calculate the transition probability in the miRNA similarity network, we make use of the Laplace normalization (Zhao et al., 2015) to calculate transition probability matrix in the miRNA similarity network, and the exit degree of nodes and the entry degree of nodes were taken into account.

Laplace normalization: Assuming that *Z*=[(*i*,*j*)],*i*,*j=*1,2,…,*N* is a symmetric matrix, Y is a diagonal matrix, defined as: *Y* (*i*,*i*) is the sum of the *i* row of *Z*, When *i* is not equal to *j*,*Y* (*i*,*j*)=0. Matrix normalization: ℤ=*Y* ^-1/2^ *AY* ^-1/2^ also a symmetric matrix, The elements in can be defined as:

$$\mathbb{Z}\left(i,j\right)=\frac{Z\left(i,j\right)}{\sqrt{Y\left(i,i\right)Y\left(j,j\right)}}$$

Then the transition probability matrix M in the miRNA similarity network can be expressed as:

$$M(i,j)=\{\begin{array}{ll} \frac{sm(i,j)}{\sqrt{\sum_{i}sm(i,j)\sum_{j}sm(i,j)}} & if\sum_{i}sm(i,j)and\sum_{j}sm(i,j)\neq 0\\ \;\;\;\;\;0 & otherwise \end{array}$$

Similarly, we can obtain the transition probability matrix D and L in the disease similarity network and lncRNA similarity network as follows:

$$D(i,j)=\{\begin{array}{ll} \frac{sd(i,j)}{\sqrt{\sum_{i}sd(i,j)\sum_{j}sd(i,j)}} & if\sum_{i}sd(i,j)and\sum_{j}sd(i,j)\neq 0\\ \;0 & otherwise \end{array}$$

$$L(i,j)=\{\begin{array}{ll} \frac{sl(i,j)}{\sqrt{\sum_{i}sl(i,j)\sum_{j}sl(i,j)}} & if\sum_{i}sl(i,j)and\sum_{j}sl(i,j)\neq 0\\ \;0 & otherwise \end{array}$$

#### TCRWMDA Algorithm Process

Specifically, TCRWMDA algorithm can be divided into two parts: one is random walk on heterogeneous networks, and the other is random walk between networks. **Table 1** introduces the process of TCRWMDA algorithm in predicting miRNA-disease association, and **Table 2** introduces the process of unbalanced random walk between networks.

**Table 1:** The description of the TCRWMDA algorithm.

Algorithm 1 TCRWMDA (Random Walk on three-layer heterogeneous network)
**Input:** Transition probability matrix M, D, L; Initial association matrix A, B, C; Parameter α, λ, β, *l, r, s.*
**Output:** Predicted miRNA-disease association matrix MD
1: MD^0^ =A/sum(A)
2: for t=1 to max (*l, r*)
3: MD'=MD
4: if *t*≤*l* then
5: $MD_{left}^{t}=\alpha \times \text{M}\times MD^{t-1}+(1-\alpha )\times [\lambda \times BNetWalk(B,\text{C,L,}\beta \text{,S})+(1-\lambda )\times A]$
6: end if *t* ≤ *r* then
7: $MD_{right}^{t}=\alpha \times MD^{t-1}\times \text{D}+(1-\alpha )\times [\lambda \times BNetWalk(B,\text{C,L,}\beta \text{,S})+(1-\lambda )\times A]$
8:$MD^{t}=\delta_{t\leq l}\times MD_{left}^{t}+\delta_{t\leq r}\times MD_{right}^{t}$
9: end for
10: return MD

E is identity matrix, if ≤*x*, δ_t≤x_ is 1, and 0 otherwise.

**Table 2:** The description of the BNetWalk algorithm.

Algorithm 2 BNetWalk (Random Walk between networks)
**Input:** Transition probability matrix L; Initial association matrix B and C; parameter β,s
**Output:** Predicted miRNA-disease association matrix R
1: ML^0^ =B/sum(B), LD^0^ =C/sum(C)
2: for t=1 to s
3: R′=*R*
4: *ML* ^t^=β ×*ML^t-1^*×*L*+(1-×β)×*B*
5: *LD* ^t^=β ×*L*×*LD^t-1^*+(1-×β)×*C*
6: R=*ML^t^*×*LD^t^*
7: end for
7: return R

##### Random Walk on Three-Layer Heterogeneous Networks

Where MD represents the predicted correlation matrix between miRNA and disease, *MD^t^* represents t-step random walk were performed MD, *A*、 *B*、 *C* denotes matrix of prior knowledge. TCRWMDA algorithm has six parameters: α, β*, l, r, s*. *l, r.* s represents the number of steps random walk on miRNA-miRNA network, disease-disease network and networks respectively. α controls network walk or return to the proportion of prior knowledge; The function of λ is to provide a new priori knowledge, there is a linearly combination of the new state form by a random walk between networks and the known initial state by λ. That is, if the current particle is in the miRNA network, then the particle has probability of α to perform the *l*-step random walk in the miRNA network, to perform the *l*-step random walk (1-α)×λ perform the s-step random walk into disease network, and has probability of (1-)×(1-λ) to return the start node. If the current particle is in the disease network, then the particle has probability of α to perform the *r*-step random walk in the disease network, has probability of (1-α)×λ perform the *s*-step random walk into miRNA network.

##### Random Walk Between Networks

ML represents the predicted association score between miRNA and lncRNA, while LD represents the probability matrix of disease generation on lncRNA. β notes the probability of controlling the random walk on the lncRNA network or returning to prior knowledge during random walk among networks. R represents the miRNA-disease association matrix formed through Random Walk between networks.

*ML^t^* and *LD^t^* represents t-step random walks were performed ML and LD, respectively. In equation (18), the association matrix between miRNA and lncRNA is multiplied by the right transition probability matrix L on the lncRNA network, which represents a random walk on lncRNA network to update ML. Similarly, the left multiplication probability transition matrix L represents a random walk on lncRNA network to update LD, finally, we can obtain association between miRNA and disease.

## Results and Analysis

### Parameter Analysis

Receiver operating characteristic curve (ROC curve) takes true positive rate (sensitivity) as the vertical coordinate and false positive rate (1-specificity) as the horizontal coordinate. The area under the ROC curve is the AUC value, which can be used as the evaluation index to intuitively evaluate the classifier. The higher the AUC value, the better the performance of the algorithm. In the process of parameter selection, AUC value is selected as the index to evaluate the influence of parameters. For an algorithm, if the parameters are set with different values, it corresponds to different models. For which model to choose, the best way is to use the model with the minimum generalization error. However, it is generally impossible to directly obtain the generalization error of the model, we select the model parameter when the AUC value is the largest.

TCRWMDA has six parameters, set step size of α, β and λ is 0.1, with values ranging from 0 to 1. For *l, r* and *s*, set the step size to 1 and the value range to 1–5. The known association between 495 miRNAs and 383 diseases verified by 5-fold cross validation. First, fix some parameters, change the value of a parameter, and then the influence of parameters on the model performance was determined according to the change of AUC value. In the process of parameter selection, the value of s was changed in the experiment, and the AUC value did not change much. The increase in the number of steps in the network could not provide us with more information, and the information that could be mined was limited. Moreover, the larger *s* was, the higher the algorithm complexity, and the performance of the model barely changed as s increased, so we set *s =* 1 in this paper, which also indicates that the data volume in the lncRNA data set is too small to provide more network structure information.

**Figure 2:**
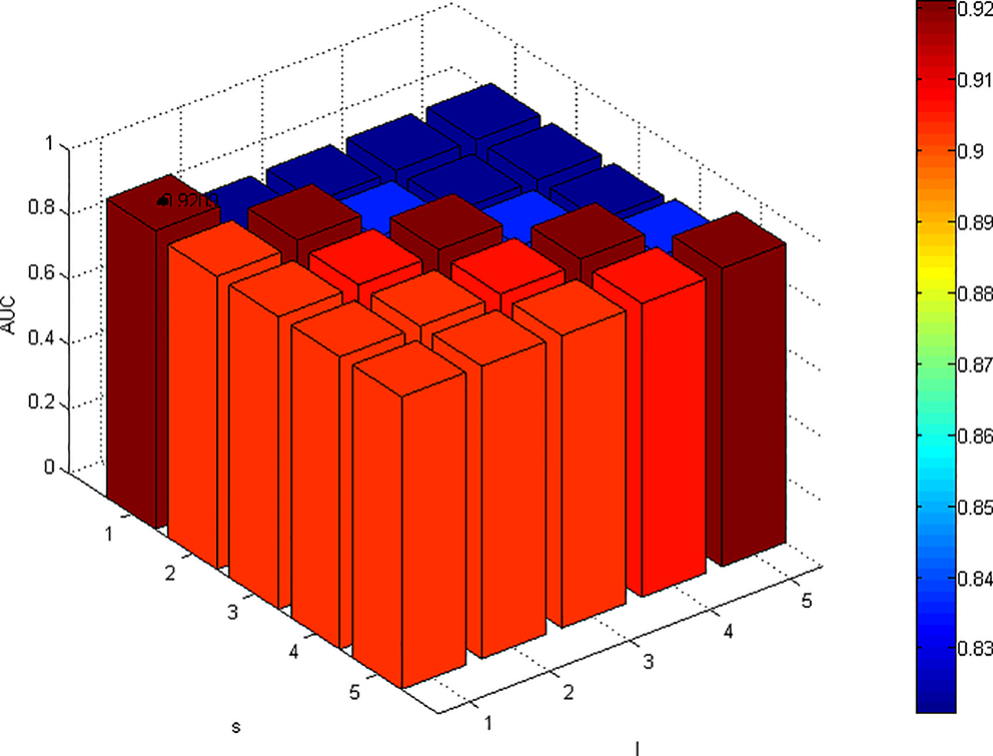
Effects of parameters l and r on the result of 5-fold cross validation. (α=0.1, β=0.1, s=1, λ=0.9). When the value of α, β, s, and λ are fixed, the AUC value is maximized when *l* and *r* are both equal to 1.

Change the values of *l* and *s* and fix other parameters. The change result of AUC is shown in **Figure 2**. For parameters *l* and *r*, the results are significantly better when *l* ≥ *r* than when *l* < *r.* Fixed *l*, with the increase of *r*, the AUC value decreased significantly, which indicated that excessive walking on the disease network would lead to a certain false positive, and the overall performance decreased. According to the results of parameter analysis, we set *l =* 1 and *r =* 1.

**Figure 3:**
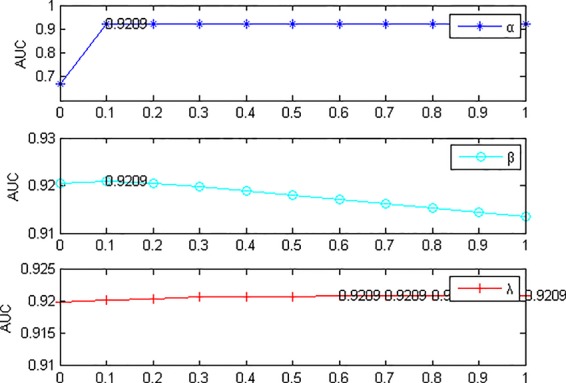
Effects of parameters α, β and λ on the result of 5-fold cross validation. (l=1, r=1, s=1). When α=0, AUC value is the lowest. In this case, only the random walk between the networks is at work. AUC is relatively stable with the variation of parameters β and λ.

Next, fix *l =* 1*, r =* 1*, s =* 1, Change the values of α, β, and λ, the experimental results are shown in **Figure 3**. α denotes restart probability, when α = 0, only random walk between networks played a role, ignoring the random walk between the miRNA network itself and the heterogeneous network on the disease network. Therefore, the results of the model were not ideal, but the remaining values of AUC were 0.9205~0.9209, with no significant fluctuation. When β = 0.1, the AUC value is the maximum and the model performance is the best. When the parameter β is larger, the probability of prior knowledge is reduced. The known association information is gradually ignored, and the results presented are reduced, which indicates that the known association information plays an important role in the algorithm itself and cannot be ignored. Parameter λ has little influence on the model, when λ = 0.9, AUC is the largest. From what has been discussed above, we select *l =* 1, *r =* 1, *s =* 1, α = 0.1, β = 0.1, λ = 0.9.

### Algorithm Performance Comparison

**Figure 4:**
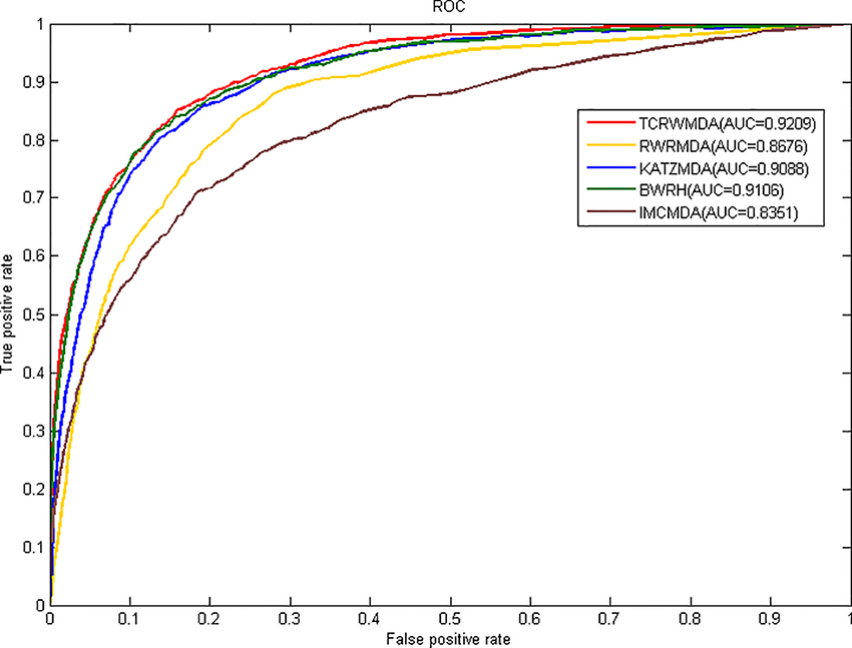
The AUC value of TCRWMDA and other methods for 5-fold cross validation.

In this paper, we take the AUC (Area under Curve) value as the evaluation index, all known miRNA-disease associations were divided into five groups of the same size, four of which were used as training set for model learning, then, the similarity calculation method mentioned above was used to calculate miRNA and disease similarity, we compare TCRWMDA with IMCMDA(Chen et al., 2018), RWRMDA(Xing Chen et al., 2012), KATZMDA (Qu et al., 2018), BRWH (Luo and Xiao, 2017) for 5-fold cross validation. The results of TCRWMDA and other methods for 5-fold cross validation are shown in **Figure 4**. True positive rate (sensitivity) is the percentage of a test sample ranked above a given threshold. False positive rate (1-specificity) is the percentage of samples below the threshold. In this paper, for the specified threshold, the true positive rate is the percentage that accurately predicts the miRNA associated with a known disease, and the false positive rate is the percentage that predicts the miRNA unrelated to the disease. When AUC = 1, the performance of the model is the best. When AUC = 0.5, it indicates that the classification method is completely ineffective and has no classification value.

It can be seen from **Figure 4**, the area under the ROC curve of TCRWMDA algorithm is the largest, that is, the prediction performance of this algorithm is better than other methods. The AUC values obtained by IMCMDA (Chen et al., 2018) (Chen et al., 2018) (Chen et al., 2018) (Chen et al., 2018) (Chen et al., 2018) (Chen et al., 2018) (Chen et al., 2018) (Chen et al., 2018) (Chen et al., 2018) (Chen et al., 2018) (Chen et al., 2018) (Chen et al., 2018), RWRMDA, KATZMDA, BRWH, and TCRWMDA on 5-fold cross validation are respectively 0.8351、0.8676、0.9088、0.9106、0.9209. The AUC value of the TCRWMDA algorithm was 1.3% higher than that of the BRWH, which indicates add new related dataset and perform a random walk on constructed multi-layer network and then is effective. TCRWMDA is 10.3% better than IMCMDA, 6.1% better than RWRMDA, and 1.1% better than KATZMDA.

### Based on Kernel-Based Soft-Neighborhood Network Fusion Similarity Model

Ma et al. considered the distance factor and the reconstruction relationship between samples to establish the nuclear soft neighborhood similarity model (Ma et al., 2018b), and combined the nuclear soft neighborhood similarity matrix of miRNA (disease) with the functional similarity (disease semantic similarity) of miRNA using similarity network fusion (SNF) (Wang et al., 2014), proposed kernel-based soft-neighborhood network fusion similarity model, and obtained good results. The following analysis based on kernel-based soft-neighborhood network fusion similarity model. After parameter analysis, the final selection is *l =* 1, *r =* 1, *s =* 1, α = 0.2, β = 0.1, λ = 0.9.

**Figure 5:**
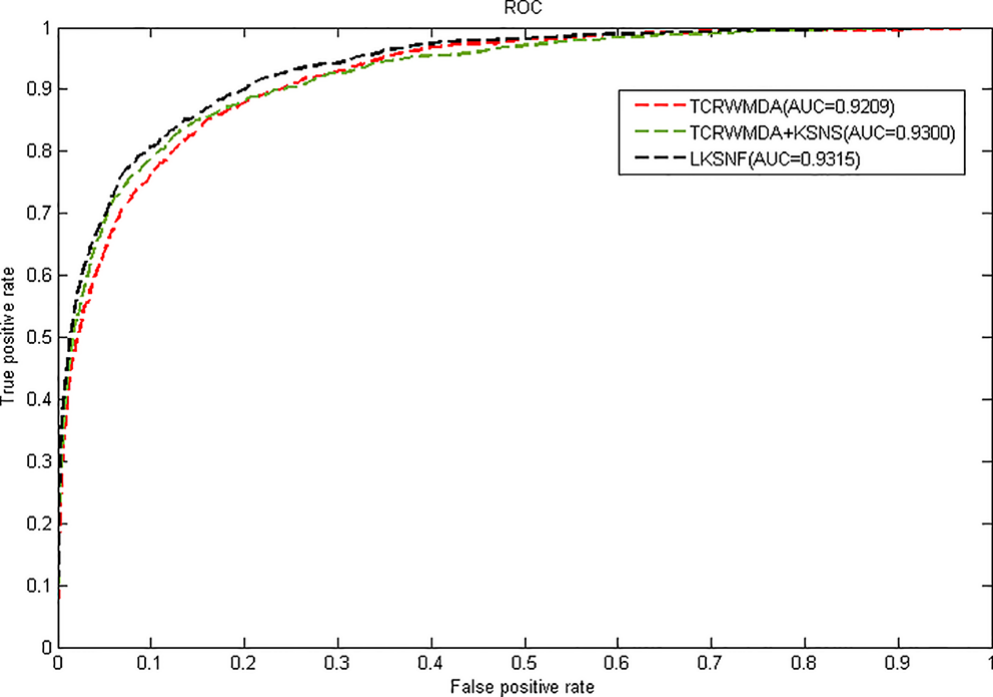
The AUC value of TCRWMDA and LKSNF for 5-fold cross validation based on kernel-based soft-neighborhood network fusion similarity model.

**Figure 5** shows the results of TCRWMDA and LKSNF soft neighborhood network of nuclear fusion based similarity model on 5-fold cross validation. In **Figure 5**, the red solid line represents the result of TCRWMDA algorithm for 5-fold cross validation, the green dotted line represents the result of TCRWMDA algorithm based on kernel-based soft-neighborhood network fusion similarity model, and the black dotted line represents the result of the LKSNF algorithm on 5-fold cross-validation. Based on kernel-based soft-neighborhood network fusion similarity model, the AUC value of the TCRWMDA algorithm is improved by 0.99%. However, the association data of lncRNA-miRNA and lncRNA-disease are sparse, the number of lncRNAs that can be considered is also small, resulting in a certain deviation in the prediction results, the AUC value obtained by TCRWMDA algorithm is almost the same as that obtained by LKSNF algorithm.

### Case Study

Globally, breast cancer is the most common cancer in women, accounting for 25% of all cancers in women. In 2012, there were 1.68 million cases of breast cancer and 520,000 deaths due to breast cancer. Mir-200c inhibits the growth and differentiation of cancer cells, and strongly inhibits the ability of normal breast stem cells to form mammary ducts and human breast cancer-driven tumorigenesis *in vivo* (Shimono et al., 2009). In addition, miRNA may be abnormally downregulated or upregulated in colon cancer tissues. In 2003, the first study on miRNAs was published in colon cancer (Michael et al., 2003), identifying mir-143 and mir-145 as new misaligned miRNAs in colon cancer.

In order to further prove the predictive performance of TCRWMDA in predicting miRNA-disease association, we used TCRWMDA algorithm to carry out analysis of breast cancer and colon cancer, as shown in **Tables 3** and **4**.

**Table 3:** The top 50 potential miRNAs predicted by TCRWMDA for breast neoplasms and their associations confirmed by database (column 1: top 1–25; Column 3: top 26–50).

miRNA	Evidence	miRNA	Evidence
hsa-mir-106a	dbDEMC	hsa-mir-454	dbDEMC
hsa-mir-130a	dbDEMC	hsa-mir-421	dbDEMC
hsa-mir-15b	dbDEMC	hsa-mir-181d	dbDEMC
hsa-mir-150	dbDEMC	hsa-mir-216a	dbDEMC
hsa-mir-192	dbDEMC	hsa-mir-330	dbDEMC
hsa-mir-142	unconfirmed	hsa-mir-451	dbDEMC
hsa-mir-130b	dbDEMC	hsa-mir-544a	dbDEMC
hsa-mir-372	dbDEMC	hsa-mir-181c	dbDEMC
hsa-mir-196b	dbDEMC	hsa-mir-198	dbDEMC
hsa-mir-98	dbDEMC	hsa-mir-376a	dbDEMC
hsa-mir-92b	dbDEMC	hsa-mir-211	dbDEMC
hsa-mir-30e	unconfirmed	hsa-mir-363	dbDEMC
hsa-mir-32	dbDEMC	hsa-mir-455	unconfirmed
hsa-mir-186	dbDEMC	hsa-mir-490	unconfirmed
hsa-mir-99b	dbDEMC	hsa-mir-494	dbDEMC
hsa-mir-424	dbDEMC	hsa-mir-381	dbDEMC
hsa-mir-212	dbDEMC	hsa-mir-154	dbDEMC
hsa-mir-449a	dbDEMC	hsa-mir-216b	dbDEMC
hsa-mir-449b	dbDEMC	hsa-mir-370	dbDEMC
hsa-mir-99a	dbDEMC	hsa-mir-520e	dbDEMC
hsa-mir-491	unconfirmed	hsa-mir-484	dbDEMC
hsa-mir-28	dbDEMC	hsa-mir-217	dbDEMC
hsa-mir-151	HMDD	hsa-mir-302e	dbDEMC
hsa-mir-144	dbDEMC	hsa-mir-590	unconfirmed
hsa-mir-95	dbDEMC	hsa-mir-377	dbDEMC

**Table 4:** The top 50 potential miRNAs predicted by TCRWMDA for colon cancer (colon neoplasms) and confirmed by database (column 1: top 1–25; Column 3: top 26–50).

miRNA	Evidence	miRNA	Evidence
hsa-mir-21	dbDEMC	hsa-mir-200a	unconfirmed
hsa-mir-20a	dbDEMC	hsa-mir-31	dbDEMC
hsa-mir-16	dbDEMC	hsa-mir-137	dbDEMC
hsa-mir-155	dbDEMC	hsa-mir-205	dbDEMC
hsa-mir-29a	dbDEMC	hsa-mir-148a	dbDEMC
hsa-mir-221	dbDEMC	hsa-mir-10b	dbDEMC
hsa-mir-143	dbDEMC	hsa-mir-125a	dbDEMC
hsa-mir-19a	dbDEMC	hsa-mir-486	dbDEMC
hsa-mir-146a	dbDEMC	hsa-let-7b	dbDEMC
hsa-mir-18a	dbDEMC	hsa-let-7f	dbDEMC
hsa-let-7a	dbDEMC	hsa-mir-375	dbDEMC
hsa-mir-200c	unconfirmed	hsa-mir-22	dbDEMC
hsa-mir-34a	dbDEMC	hsa-mir-24	dbDEMC
hsa-mir-92a	dbDEMC	hsa-mir-27a	dbDEMC
hsa-mir-9	dbDEMC	hsa-mir-214	dbDEMC
hsa-mir-222	dbDEMC	hsa-mir-183	dbDEMC
hsa-mir-125b	dbDEMC	hsa-mir-18b	dbDEMC
hsa-mir-196a	dbDEMC	hsa-mir-140	dbDEMC
hsa-let-7c	dbDEMC	hsa-mir-7	dbDEMC
hsa-mir-107	dbDEMC	hsa-mir-142	unconfirmed
hsa-let-7e	dbDEMC	hsa-let-7i	dbDEMC
hsa-mir-141	dbDEMC	hsa-mir-25	dbDEMC
hsa-mir-106b	dbDEMC	hsa-mir-199a	unconfirmed
hsa-mir-93	dbDEMC	hsa-mir-133b	dbDEMC
hsa-mir-223	unconfirmed	hsa-mir-29c	dbDEMC

The predicted results were verified by dbDEMC database (Yang et al., 2017) and HMDD (Li et al., 2013), for breast tumor diseases, 44 of the first 50 predicted miRNAs were verified in dbDEMC and 45 of the top 50 predicted colon tumor diseases were verified by dbDEMC. In order to enhance the persuasion, we also listed two other cases (lung neoplasms and lymphoma), whose prediction results were verified as shown in the **Supplementary Tables 1** and **2**.

## Conclusion

With the development of bioinformatics, more and more experiments and evidence show that miRNA is closely related to the generation and development of human diseases, and the discovery of miRNA that may be related to diseases has attracted much attention. The experiment is time-consuming and costly, the new and effective miRNA-disease association prediction algorithm can effectively provide research directions and reduce the cost and time of biological experiments.

In this paper, we propose a novel TCRWMDA algorithm, which is different from the traditional prediction methods based on heterogeneous network and incorporates new prior knowledge (lncRNA information related to miRNA and disease) to effectively make the best use of the information that we have. TCRWMDA is a framework for integrating multiple sources of information, which may yield better results when the data set is large. TCRWMDA is applied to miRNA-disease association prediction, which implements unbalanced random walk on three-layer heterogeneous networks and integrate the related similarity information to predict disease-related miRNAs. TCRWMDA is efficient because it makes use of multi-source information from reliable data sources. Considering the association between lncRNA and disease and the association between miRNA and disease, TCRWMDA mines the association information on between data and topological information in the network to improve the prediction accuracy. Experimental results and case studies prove that the TCRWMDA algorithm is an effective tool for predicting the potential miRNA-disease association. If more data sets are added, the increase and optimization of parameters is a problem worth thinking about. In the future, we hope to conduct more stable data integration and seek methods for optimizing parameter selection.

## Data Availability Statement

All datasets for this study are included in the article/**Supplementary Material**.

## Author Contributions

LY and XS designed and implemented the computing framework. LY and XS analyzed the results and wrote the manuscript. LY, XS, DZ and JY revised the manuscript. LY prepared the computational codes and carried out. All the authors wrote, reviewed and approved the final manuscript.

## Funding

This research was supported by the National Natural Science Foundation of China (61532008, 61872157, 61932008), the Self-determined Research Funds of CCNU from the Colleges' Basic Research and Operation of MOE (CCNU19QD003) and the National Language Commission Key Research Project (ZDI135-61).

## Conflict of Interest

The authors declare that the research was conducted in the absence of any commercial or financial relationships that could be construed as a potential conflict of interest.
