# Supplementary material for: Three-Layer Heterogeneous Network Combined With Unbalanced Random Walk for miRNA-Disease Association Prediction
Source: Front Genet. 2020 Jan 10;10:1316. doi: 10.3389/fgene.2019.01316 (PMC6967737; doi:10.3389/fgene.2019.01316)
Supplement: Supplementary file 1 [file Table_1.pdf]

Supplementary Material

1 Supplementary Tables

Table 1. The top 50 potential miRNAs predicted by TCRWMDA for lung neoplasms and confirmed by database (column 1: top 1-25; column 3: top 26-50).

miRNA	Evidence	miRNA	Evidence
hsa-mir-16	dbDEMC	hsa-mir-302d	dbDEMC
hsa-mir-106b	dbDEMC	hsa-mir-99b	dbDEMC
hsa-mir-15a	dbDEMC	hsa-mir-215	dbDEMC
hsa-mir-141	dbDEMC	hsa-mir-367	dbDEMC
hsa-mir-20b	dbDEMC	hsa-mir-424	dbDEMC
hsa-mir-15b	dbDEMC	hsa-mir-10a	dbDEMC
hsa-mir-195	dbDEMC	hsa-mir-449a	dbDEMC
hsa-mir-130a	dbDEMC	hsa-mir-122	dbDEMC
hsa-mir-429	dbDEMC	hsa-mir-28	dbDEMC
hsa-mir-302b	dbDEMC	hsa-mir-99a	dbDEMC
hsa-mir-193b	dbDEMC	hsa-mir-449b	dbDEMC
hsa-mir-373	dbDEMC	hsa-mir-144	dbDEMC
hsa-mir-302a	dbDEMC	hsa-mir-181d	dbDEMC
hsa-mir-296	unconfirmed	hsa-mir-342	dbDEMC
hsa-mir-23b	dbDEMC	hsa-mir-491	dbDEMC
hsa-mir-92b	dbDEMC	hsa-mir-488	dbDEMC
hsa-mir-204	dbDEMC	hsa-mir-520c	unconfirmed
hsa-mir-194	dbDEMC	hsa-mir-129	dbDEMC
hsa-mir-130b	dbDEMC	hsa-mir-149	dbDEMC
hsa-mir-302c	dbDEMC	hsa-mir-383	dbDEMC
hsa-mir-339	dbDEMC	hsa-mir-520d	dbDEMC
hsa-mir-196b	dbDEMC	hsa-mir-139	dbDEMC
hsa-mir-372	dbDEMC	hsa-mir-301b	dbDEMC
hsa-mir-153	dbDEMC	hsa-mir-151	unconfirmed
hsa-mir-520b	dbDEMC	hsa-mir-148b	dbDEMC

Table 2. The top 50 potential miRNAs predicted by TCRWMDA for lymphoma and confirmed by database (column 1: top 1-25; column 3: top 26-50).

miRNA	Evidence	miRNA	Evidence
hsa-mir-34a	dbDEMC	hsa-mir-199a	dbDEMC
hsa-mir-223	dbDEMC	hsa-let-7i	dbDEMC
hsa-mir-125b	dbDEMC	hsa-mir-148a	dbDEMC
hsa-mir-221	dbDEMC	hsa-let-7g	dbDEMC
hsa-mir-29b	dbDEMC	hsa-mir-34c	unconfirmed
hsa-let-7a	dbDEMC	hsa-mir-141	dbDEMC
hsa-mir-222	dbDEMC	hsa-mir-196a	dbDEMC
hsa-mir-106b	dbDEMC	hsa-mir-15b	dbDEMC
hsa-mir-145	dbDEMC	hsa-mir-142	unconfirmed
hsa-mir-29a	dbDEMC	hsa-mir-181b	dbDEMC
hsa-mir-9	dbDEMC	hsa-mir-34b	dbDEMC
hsa-mir-93	dbDEMC	hsa-mir-182	dbDEMC
hsa-mir-214	dbDEMC	hsa-mir-7	dbDEMC
hsa-let-7b	dbDEMC	hsa-mir-130a	dbDEMC
hsa-mir-143	dbDEMC	hsa-mir-27a	dbDEMC
hsa-mir-106a	dbDEMC	hsa-mir-10b	dbDEMC
hsa-mir-205	dbDEMC	hsa-mir-30c	dbDEMC
hsa-let-7f	dbDEMC	hsa-mir-196b	dbDEMC
hsa-mir-195	dbDEMC	hsa-mir-25	dbDEMC
hsa-let-7d	dbDEMC	hsa-mir-1	dbDEMC
hsa-let-7e	dbDEMC	hsa-mir-302b	dbDEMC
hsa-let-7c	dbDEMC	hsa-mir-193b	dbDEMC
hsa-mir-146b	dbDEMC	hsa-mir-130b	dbDEMC
hsa-mir-183	dbDEMC	hsa-mir-100	dbDEMC
hsa-mir-31	dbDEMC	hsa-mir-335	dbDEMC
